# Modifications to gene body methylation do not alter gene expression plasticity in a reef‐building coral

**DOI:** 10.1111/eva.13662

**Published:** 2024-02-22

**Authors:** Evelyn Abbott, Coral Loockerman, Mikhail V. Matz

**Affiliations:** ^1^ Department of Integrative Biology University of Texas at Austin Austin Texas USA; ^2^ University of Hawai'i at Manoa Hawaii Institute of Marine Biology Kaneohe Kaneohe Hawaiʻi USA

**Keywords:** adaptation, climate change, epigenetics, phenotypic plasticity, transcriptomics

## Abstract

As coral reefs continue to decline due to climate change, the role of coral epigenetics (specifically, gene body methylation, GBM) in coral acclimatization warrants investigation. The evidence is currently conflicting. In diverse animal phyla, the baseline GBM level is associated with gene function: continuously expressed “housekeeping” genes are typically highly methylated, while inducible context‐dependent genes have low or no methylation at all. Some authors report an association between GBM and the environment and interpret this observation as evidence of the GBM's role in acclimatization. Yet, others argue that the correlation between GBM change and gene expression change is typically absent or negligible. Here, we used the reef‐building coral, *Acropora millepora,* to test whether environmentally driven changes in GBM are associated with a gene's ability to respond to environmental changes (plasticity) rather than expression level. We analyzed two cases of modified gene expression plasticity observed in a 3‐week‐long heat acclimatization experiment. The first one was a group of heat‐induced genes that failed to revert their expression after the coral was translocated back to the control tank. The second case involved genes that changed the magnitude of their response to the daily temperature fluctuations over the course of the experiment. In both cases, we found negligible or no association with GBM change. We conclude that although both gene expression plasticity and GBM can change during acclimatization, there is no direct association between the two. This adds to the increasing volume of evidence that the function of GBM in invertebrates is unrelated to acclimatization on physiological timescales.

## 1 INTRODUCTION

Ocean warming due to anthropogenic climate change has caused increasingly consistent, severe coral bleaching events, leading to widespread reef decline. There has been a growing interest in epigenetics and how it may facilitate adaptation in a rapidly changing environment. However, as we will discuss here, there is conflicting evidence that modifications to the epigenome serve an adaptive function in cnidarians (Dixon & Matz, 2022; Harris et al., 2019; Liew et al., 2018, 2020).

DNA methylation is a common epigenetic modification among eukaryotes, which may have originated as a mechanism for genomic defense against transposable elements (Chandler & Walbot, 1986; Hackett & Surani, 2013). In animals, it is highly heritable (Bird, 2002) and maintained during cell division by DNA methyltransferases, which are homologous among vertebrates and invertebrates (Lyko, 2018). DNA methylation in vertebrates occurs throughout the genome, including promoters, enhancers, and gene bodies. Promoter methylation serves a variety of functions, including gene silencing, X‐inactivation, gene imprinting, and cell differentiation, during development (Moore et al., 2013; Smith & Meissner, 2013). Methylation of gene bodies, on the other hand, is linked to elevated gene expression (Jones, 2012).

In invertebrates, DNA methylation levels vary greatly between taxa and occurs almost exclusively in gene bodies (Suzuki et al., 2007). Gene body methylation (GBM) level has a bimodal distribution across the genome and is correlated with how consistently a gene is expressed. Genes with high GBM are constitutively expressed, while genes with low GBM are dynamically regulated and tend to be expressed at lower levels (Jones, 2012; Suzuki et al., 2007; Zemach et al., 2023). This raises the question of whether the environment can change GBM and if this change can alter gene expression.

Previous studies on many invertebrates have demonstrated that GBM may be subject to change, including species of bees, ants, moths, and crayfish (de Mendoza et al., 2020; Gatzmann et al., 2018; Harris et al., 2019). This has also been demonstrated in cnidarians, such as anemones and corals (Dixon & Matz, 2022). However, the potential function of these changes remains controversial. One study which investigated ocean acidification acclimatization in the coral *Stylophora pistillata* found that higher‐intensity stress was associated with increased GBM and decreased transcriptional noise (Liew et al., 2018). Another study found that in the coral *Platygyra daedalea* GBM patterns were associated with specific life cycle stages and that GBM changes were associated with offspring survival (Liew et al., 2020).

On the contrary, other studies have come to different conclusions. A study of the crayfish, *Procambarus virginalis*, found no variation in GBM across tissue types (Gatzmann et al., 2018), despite profound differences in gene expression. Another study conducted on European honey bees found that while GBM varies during development, this variability has no association with gene expression change (Harris et al., 2019). In other insects, DNMT gene knockdown lineages had no transcriptional differences compared to their wild‐type counterparts (de Mendoza et al., 2020). Furthermore, a meta‐analysis spanning eight invertebrate taxa, including two cnidarians (the coral *Stylophora pistillata* and the anemone *Exaiptasia pallida*), found no biologically relevant correlation between GBM change and gene expression (Dixon & Matz, 2022).

Here, we hypothesized that environmentally driven GBM changes may not affect the level of gene expression, but rather the plasticity of gene expression, i.e., the ability of a gene to change its expression in response to environmental perturbation. Specifically, we envisioned that modification of GBM during acclimatization might prevent genes that changed their expression from reverting back to their original state. Our hypothesis was that acclimatization‐related GBM change would influence gene expression plasticity, which could be a mechanism by which the organism could “learn” to maintain certain gene expression levels despite short‐term environmental fluctuations. To test this hypothesis, we conducted a 2‐week heat acclimatization experiment with the reef‐building coral *Acropora millepora* to induce GBM change. We then moved a subset of the heated corals to the control condition for a day to test whether genes with changed GBM remained in the “heated” state. Because high GBM is observed in genes with more stable expression, we expected that increasing methylation would reduce gene expression plasticity.

## 2 MATERIALS AND METHODS

### 2.1 Sample sourcing and experimental design

**FIGURE 1:**
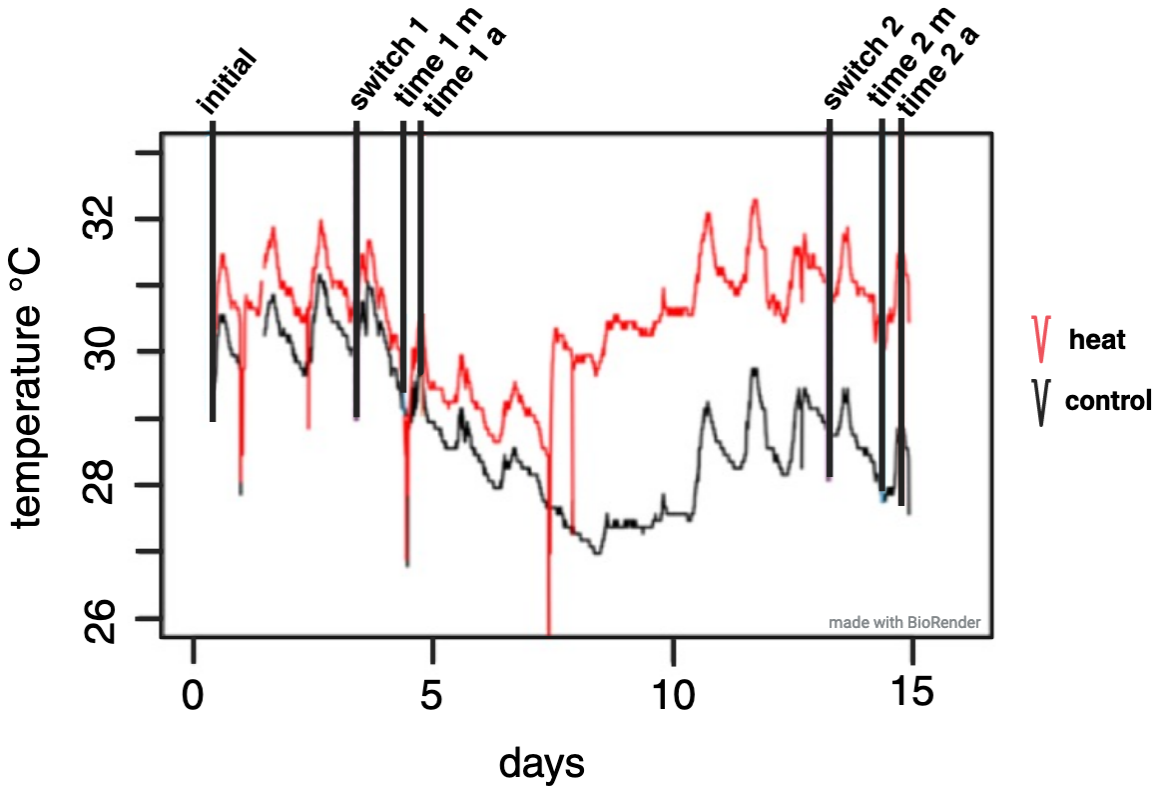
Temperatures (y‐axis) of the control (black line) and heat (red line) container during the experiment (x‐axis). Vertical lines represent the sampling time points: switch time point 1 (switch 1), time point 1 morning (time 1 m), time point 2 afternoon (time 1 a), switch time point 2 (switch 2), time point 2 morning (time 2 m), and time point 2 afternoon (time 2 a).

The experiment was conducted at Orpheus Island Research Station in November 2018 under GBRMPA permit G18/41245.1. Three *Acropora millepora* colonies were collected by SCUBA at Northeast Orpheus and placed in raceways with unfiltered seawater. Colonies were selected by two criteria. First, they must be adults and at least 30 cm across. Second, we only selected healthy colonies, using lack of bleaching and the presence of eggs as a proxy. The experiment was started 5 days after spawning. The temperatures and experimental time points are shown in Figure 1. The corals were then fragmented into 5–7‐cm fragments containing two–three tips (“twigs”) and returned to the raceway. For the experiment, twigs were hung from a fishing line in two 70‐L containers with a slow flow‐through of filtered seawater and aeration to create water movement. To simulate the natural, daily variation in temperature of coral reef ecosystems, this experiment was conducted on shaded tables outside. This resulted in temperature variation in both heat and control containers. At the start of the experiment, a heater was added to the heat container and was set to 29.5°C. Due to unexpected warm weather, on day 7, the heater temperature was raised to 31°C to create a greater contrast with the control temperature.

On day 4, 12 samples from the heat group were switched to the control container and allowed to acclimatize for 24 h. At 8:00 a.m. on day 5, six samples were taken from each of the heat, control, and switch groups, followed by another six samples from each group at 4:00 p.m. This process was repeated on day 13 and for another 12 samples from all groups on day 14, six from each at 8:00 a.m. and 4:00 p.m. Samples were immediately fixed in 100% ethanol and stored at −80°C.

### 2.2 Nucleic acid extraction and library preparation

RNA was extracted using an RNAqueous total RNA isolation kit. Samples were submerged in a lysis buffer and pulverized using 150–212 μM glass beads and Biospec Mini‐Beadbeater for 20 s. The RNA was then extracted following the kit's protocol. DNA was isolated from the same lysate using a phenol:chloroform:isoamyl alcohol extraction, followed by purification with a Zymo cleanup and concentrator kit. Details of the DNA extraction protocol can be found at https://github.com/evelynabbott/DNA_methylation_plasticity. TagSeq libraries for gene expression analysis (Meyer et al., 2011) were prepared according to the protocol hosted on the TagSeq github page, https://github.com/z0on/tag‐based_RNAseq. Gene body methylation (GBM) was analyzed using the mdRAD protocol, which uses methylation‐dependent restriction enzymes to target methylated DNA (Dixon & Matz, 2021). After sequencing, the methylation level is determined by the number of reads mapping to the given region. The mdRAD library preparation protocol can be found at the github page, https://github.com/evelynabbott/DNA_methylation_plasticity. BluePippin (Sage Science) was used to size select for fragments 350–550 bp in length, and all samples were sequenced at a depth of 2–3 million reads per sample. Sequencing was conducted at the Genome Sequencing and Analysis Facility at the University of Texas at Austin.

### 2.3 Data processing

**FIGURE 2:**
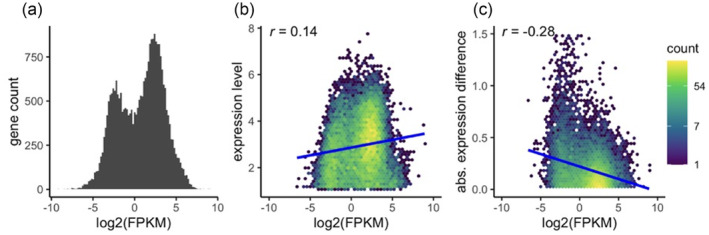
Relationship between gene expression and GBM level across all samples, quantified by reads per kilobase of exon per million reads, log_2_(FPKM). (a) Distribution of GBM levels across all genes. (b) Relationship between GBM level and gene expression level. (c) Relationship between GBM level and magnitude of gene expression change between experimental time points.

Details of the data processing pipeline and scripts used can be found at https://github.com/evelynabbott/DNA_methylation_plasticity. Adapters were trimmed in single‐end mode using cutadapt (Martin, 2011), with a minimum length of 20 bp and a PHRED quality cutoff of 20. FASTQC (Andrews, 2010) was used to check the quality of a subset of 10,000 reads before and after trimming. Reads were mapped to the *Acropora millepora* reference genome (Fuller et al., 2019) using bowtie2 with the ‐‐local option, and PCR duplicates were identified using MarkDuplicates from the Picard toolkit (Broad Institute, 2019). Sam files were sorted and converted to bam files using Samtools (Li et al., 2009). mdRAD reads mapping to annotated gene boundaries were counted using FeatureCounts (Liao et al., 2014), and BEDTools Multicov was used to determine fold coverage in different region types (such as transcriptional start sites, promoter boundaries, and gene boundaries). The number of mdRAD counts per sample ranged from 569,974 to 1,321,009, and the number of TagSeq counts per sample ranged from 235,588 to 825,559. The results from BEDTools Multicov were used for mdRAD statistical analyses. Fragments Per Kilobase of transcript per Million mapped reads (FPKM) averaged across all samples was used to calculate the methylation level for each gene (Figure 2a–c). FPKM was used to visualize the characteristic bimodal distribution of GBM observed in previous studies and to confirm that high GBM and low GBM were correlated with constitutive and plastic expression, respectively (e.g., Dixon et al., 2014; Sarda et al., 2012). FPKM was averaged across all samples because we were concerned with the correlation between baseline methylation and gene expression, rather than methylation change.

### 2.4 GBM level and its association with gene expression

Before analyzing the effects of GBM change on gene expression, we first sought to confirm that our results were consistent with findings from similar studies. In invertebrates, GBM has a bimodal distribution throughout the genome (Dixon et al., 2014). Moreover, it tends to be high in genes which are constitutively and highly expressed and low in genes which are expressed at lower levels and are dynamically regulated (Sarda et al., 2012). As expected, we found the characteristic bimodal distribution of GBM levels among genes (Figure 2a). There was a positive correlation between baseline GBM level and gene expression (*r* = 0.14, *p* = 1.99e‐63; Figure 2b) and a negative correlation between GBM and gene expression plasticity between experimental time points (*r* = −0.28, *p* = 1.77e‐276; Figure 2c).

### 2.5 Differential gene expression and methylation analysis

To investigate the relationship between gene expression and GBM change due to treatment, experimental time point, and time of day, TagSeq and mdRAD counts were imported into DESeq2 (Love et al., 2014) to generate normalized variance‐stabilized data. To compute log_2_ fold differences, treatment (control, heat, or switch), experimental time point (1 or 2), and time of day (morning or afternoon) were used as predictive variables.

This DESeq2 formula was also used to analyze the relationship between daily plasticity and GBM change. To compute daily plasticity at timepoints 1 and 2, log_2_ fold expression changes due to the time of day were computed separately for each timepoint. DESeq2 was then repeated with all the samples combined, but with the addition of the interaction between timepoint of day and experimental time point. The log_2_ fold changes computed from the interaction term represent the change in daily plasticity over time.

Contour lines representing the effect of GBM log_2_ fold changes and GBM base mean (the average of normalized count values, divided by size factors, across all samples) on gene expression were calculated using the function *ordisurf()* from the R package *vegan* (Oksanen, 2010). *ordisurf()* fits ordination plots with contour lines representing the effect of a given variable using generalized additive modeling.

### 2.6 Confirmation that heat treatment elicits distinct gene expression response

**FIGURE 3:**
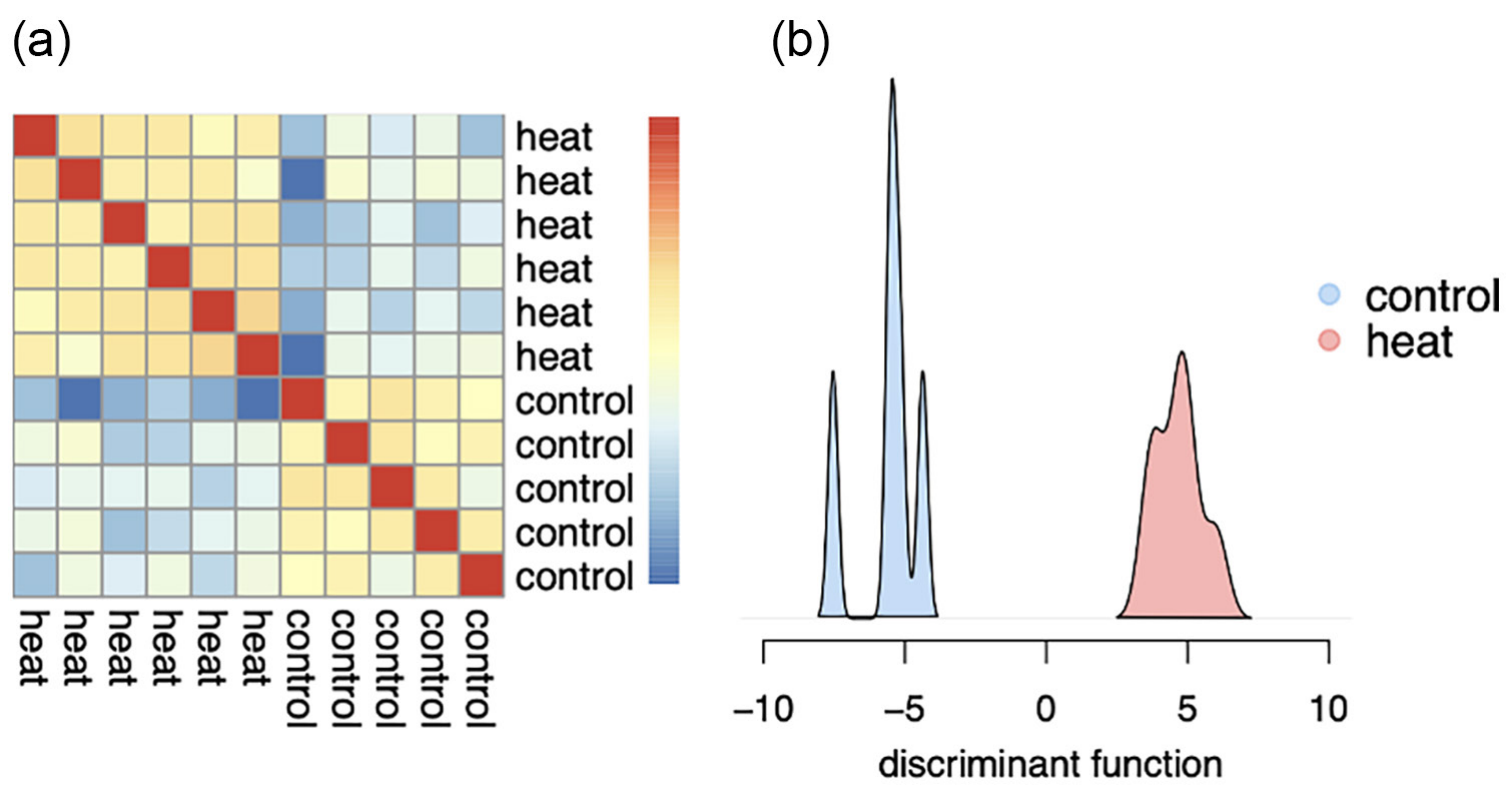
Broad patterns of gene expression in heat and control groups in the morning at timepoint 2. Figures including afternoon and timepoint 1 samples can be found in Figure S2. (a) Heatmap showing similar (red) and different (blue) gene expression between samples. The color represents the correlation coefficient on a scale of 1 (red) to 0 (blue). (b) Results of DAPC. Colors represent heat group (red) and control group (blue) gene expression. (Morning samples only).

Heatmaps showing broad patterns of gene expression were generated using the variance stabilized counts from DESeq2. The data were plotted using the function *pheatmap::pheatmap*, clustering genes (rows) into 250 groups of similar expression using the k‐means algorithm. Heatmaps were generated for timepoints 1 and 2 separately, though they showed a small structure depending on treatment when afternoon samples were included, likely due to similarities between heat treatment and peak afternoon temperatures (Figure S1). When separated by timepoint and the time of the day, it is visually clear that treatment was the predominant driver of gene expression at time point 2 in the morning samples (Figure 3a).

Differences between treatment groups, timepoints, and the time of the day were also investigated using discriminant analysis of principal components (DAPC). DAPC was plotted for each timepoint and time of day individually (Figure S2). In accordance with the heatmaps, the difference between heat and control samples was most apparent in the morning samples at the second timepoint (Figure 3b) Because of these results, only morning samples were used in treatment analyses in the main text. Analyses including afternoon samples can be found in the supplemental information (Figures S2 and S3).

### 2.7 Weighted gene co‐expression network analysis (WGCNA)

**FIGURE 4:**
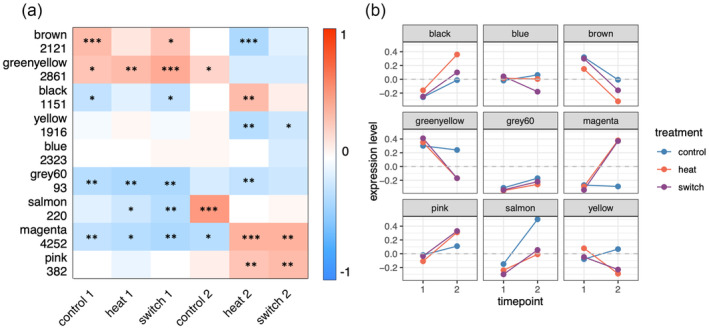
Expression of WGCNA modules in all treatment groups (control, heat, or switch) and timepoints (1 or 2). (a) Heatmap showing the correlation coefficient between module eigengenes and treatment groups. Red indicates a positive relationship, and blue indicates a negative relationship. Row names correspond to module names and the number of genes in the module. Asterisks correspond to significance: *p ≤ 0.05, **p ≤ 0.01, ***p ≤ 0.001. A heatmap including exact R and *p*‐values can be found in Figure S4. (b) Module gene expression level of each treatment group. Y‐axis positive values indicate upregulation, and negative values indicate downregulation. X‐axis represents experimental timepoints 1 and 2. Lines represent treatment groups, control (blue), heat (red), and switch (purple). (Morning samples only).

To identify modules of genes co‐regulated due to treatment in the morning samples, we used WGCNA (Langfelder & Horvath, 2008). The input for this analysis was a matrix of variance‐stabilized counts generated by DESeq2 with the effect of the genotype removed using the R package limma. Soft threshold was set at 12, and the cut height was set at 0.4, generating nine modules (Figure 4a,b).

Reduced plasticity of these modules was determined by the directionality of expression (up vs downregulation) of the treatment groups based on two criteria: first, prior to switching from the heat to the control condition (first timepoint), the directionality of expression in the switch and control groups must be the same and statistically significant (*p* < 0.05). Second, after switching from the heat to control condition (second timepoint), the directionality in the switch group must be the same as that of the heat group and the opposite of the control group. This would indicate that after acclimatizing for 24 h, the switched expression remained in the heated state rather than reverting to pre‐treatment, control state.

One module, “magenta” (4252 genes), was determined to have reduced plasticity in the switch group at the second timepoint as per the above criteria (Figure 4a,b). Another module, “green‐yellow” (2861 genes) also had reduced plasticity in the switch group at the second timepoint, although this was not statistically significant in the heat and switch groups at time point 2 (Figure 4a,b).

## 3 RESULTS

### 3.1 Quantifying generalized stress response (GSR)

**FIGURE 5:**
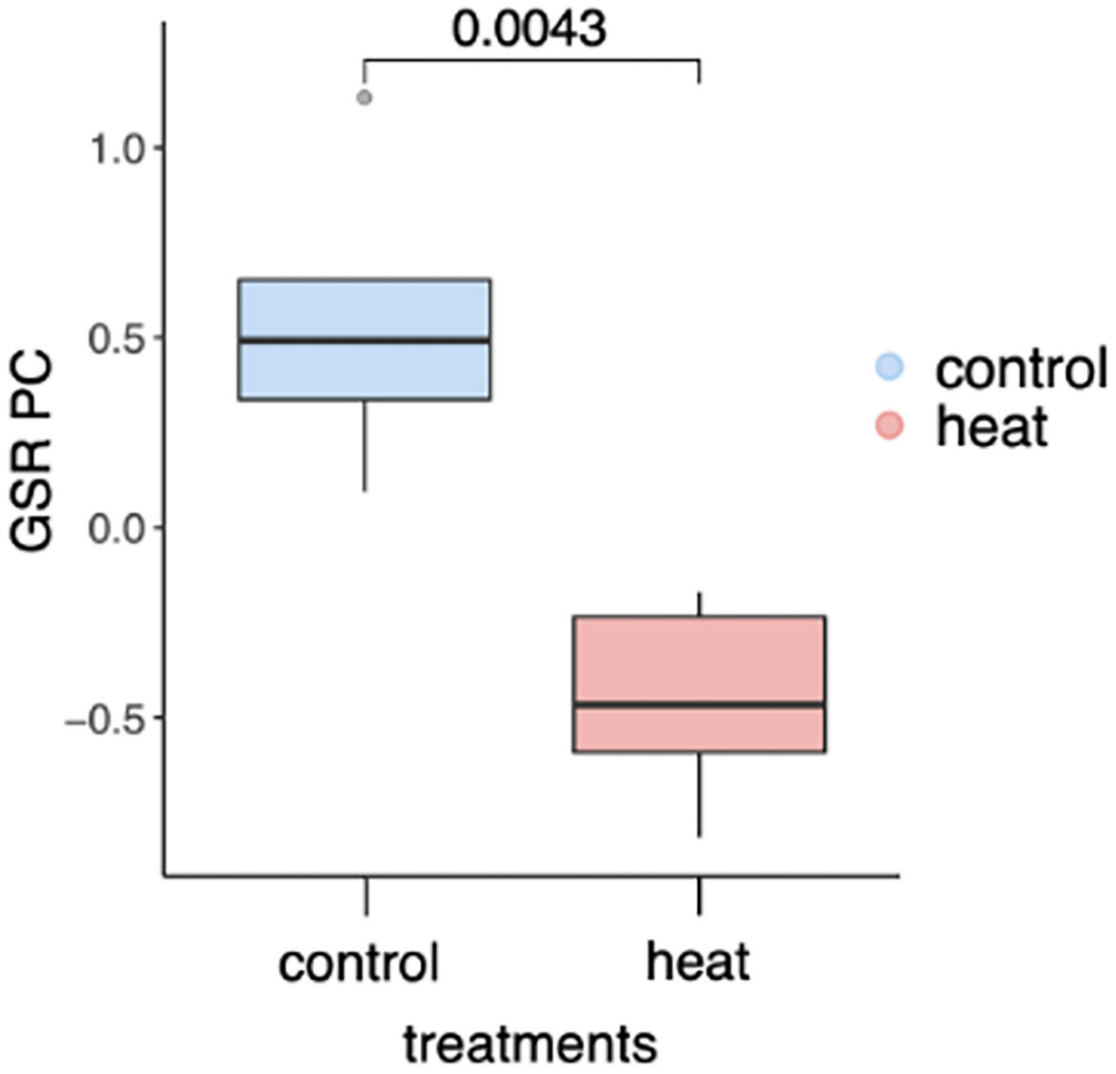
Expression of generalized stress response genes (“red module”) in control (blue) and heated samples (red) in the morning at the second timepoint.

To determine if heat treatment induced a stress response in the morning samples, we quantified the generalized stress response represented by the “red module”, a group of 634 genes which are upregulated in response to any bleaching‐level stress in *Acropora* sp. (Dixon et al., 2020). Conversely, in response to lower‐intensity stress, the red module is downregulated. We computed the eigengene of the module, which is defined as the first principal component of all genes' expression variation in a module and represents the gene expression profile of a module (Langfelder & Horvath, 2008). We found that at the second timepoint, this module was significantly downregulated in heated samples compared to controls (Figure 5, *r* ^2^ = 0.004), indicating that the corals were experiencing low‐intensity stress.

### 3.2 Broad patterns of gene expression and GBM log‐fold change

**FIGURE 6:**
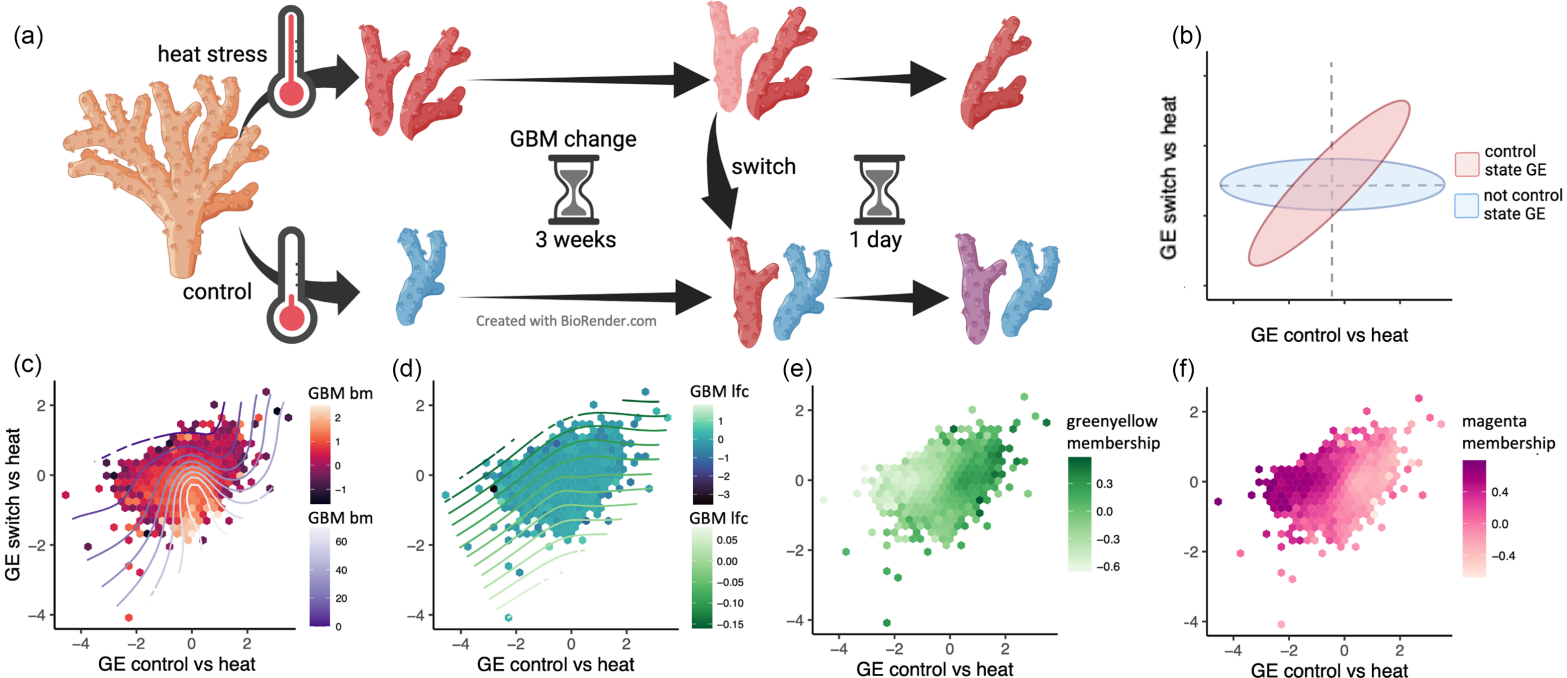
Correlation between control and switch gene expression compared to the heat group at the end of the experiment. (a) Experimental design showing the control, heat, and switch groups. (b) Hypothetical results showing control state gene expression (horizontal ellipse), non‐control state gene expression (diagonal ellipse), and unchanged gene expression (intersection of both ellipses). (c–f) Experimental results showing the correlation between control and switch gene expression at the end of the experiment. Hexagons represent groups of genes binned together due to similar expression. Color of the hexagons and contour lines correspond to GBM base mean (c, *r* ^2^ = 0.058), GBM lfc (*r* ^2^ = 0.002), membership in the green‐yellow module (e), and membership in the magenta module (f). (Morning samples only).

Using normalized variance‐stabilized gene expression data from DESeq2, we looked for genes in the morning samples whose expression remained in the “heat state” 24 h after the fragment was switched from the heat tank to the control tank (red ellipse in Figure 6b). We then compared the GBM of these genes to those whose expression reverted to the “control state” after switching (blue ellipse in Figure 6b). Our hypothesis was that acclimatization‐related GBM change would influence the gene expression plasticity, which could be a mechanism by which the organism could “learn” to maintain certain gene expression levels despite short‐term environmental fluctuations.

Overall, most of the genes in the switch group reverted to the control state (*r* ^2^ = 0.135, *p* < 2.2e‐16). However, there were also many genes that fell into the “red ellipse” region of reduced plasticity (Figure 6b). To better visualize the relationship between GBM and plasticity, we used the ordisurf() function from the R package vegan, which fits smooth surfaces for continuous variables over ordination plots. We used GBM results from the DESeq2 model to plot contour lines representing the effect of GBM log_2_fold change and baseline GBM. Baseline GBM explained a small fraction of the variance in gene expression (Figure 6c, *r* ^2^ = 0.058), as expected from the general association of baseline GBM with gene expression variability (Figure 6c). Baseline GBM peaked in genes that reverted after switching but were not differentially expressed between the heat and control groups; it did not show peaks or troughs in the areas of the plot where genes with reduced gene expression plasticity were found (Figure 6c). GBM log_2_fold change showed no association with any area of the plot and had only a negligible correlation with gene expression change (Figure 6d, *r* ^2^ = 0.002).

### 3.3 Coexpression module with reduced plasticity

In addition to qualitatively designating genes as high or low plasticity based on their position in the scatter plot (Figure 6b), we have applied an unsupervised gene clustering method WGCNA (Langfelder & Horvath, 2008) to group genes into coexpressed modules and looked for modules that show signatures of reduced plasticity, i.e., differing between heat and control but not between heat and switch groups. We identified two such modules, “magenta” and “green‐yellow” (4252 and 2861 genes, respectively, Figure 4).

**FIGURE 7:**
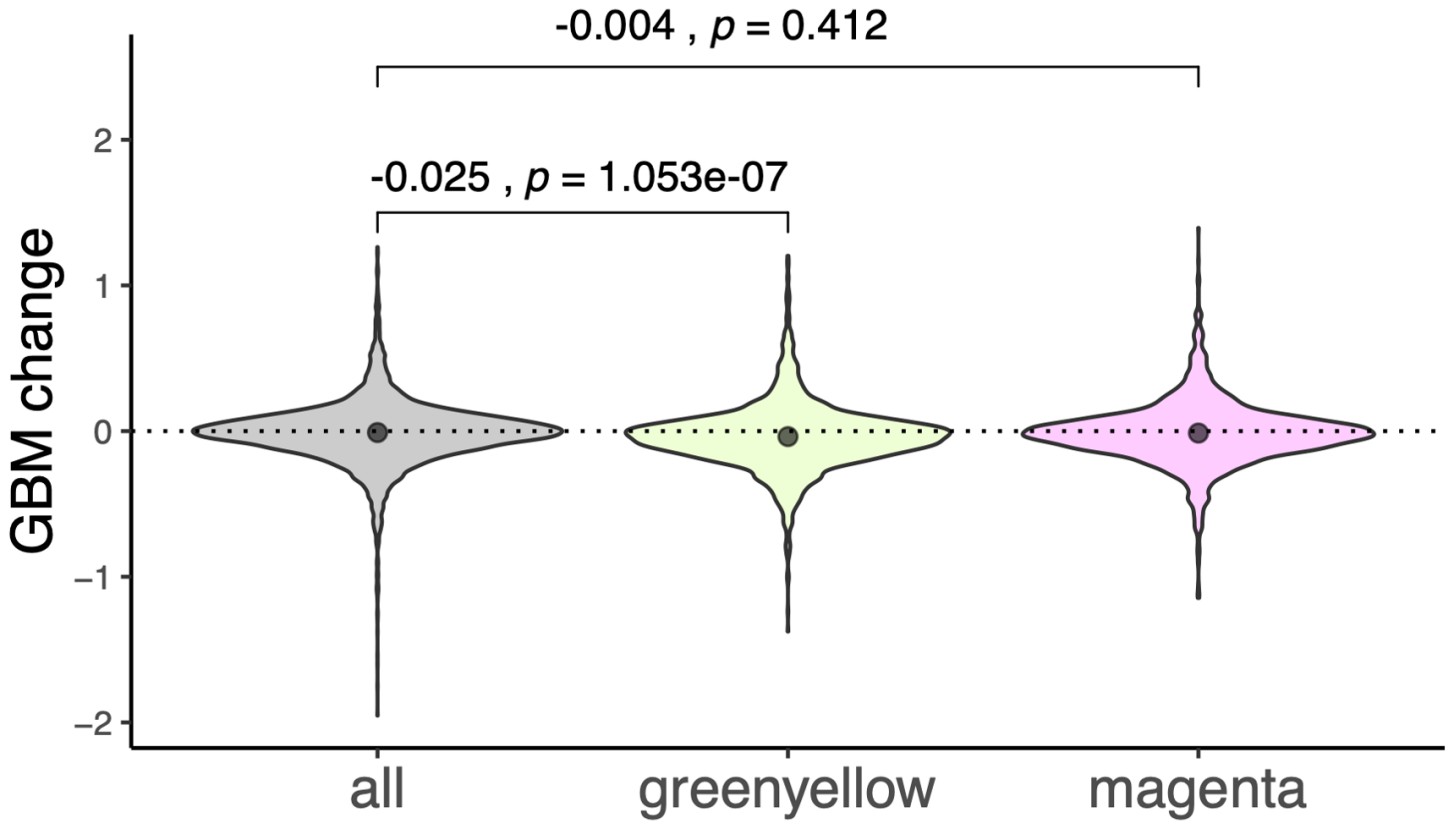
GBM log_2_fold change between timepoints 1 and 2 across all genes (left), the green‐yellow module (center), and the magenta module (right). Numbers above violin plots indicate the effect size and significance (left and right numbers on each bracket, respectively) of the mean GBM change in all, magenta, and green‐yellow genes. A positive value indicates increase in methylation, and a negative value indicates a decrease in methylation. (Morning samples only).

Genes with a high magenta module membership score and low green‐yellow membership score (quantified by kME, the correlation of the gene's expression with the module's eigengene) formed the core of the “reduced plasticity” region of the scatter plot (Figure 6b,e,f). Detecting this large group of coexpressed genes in an unsupervised fashion suggests that some common regulatory mechanism was responsible for their reduced plasticity in our experiment. We next compared the GBM change between time points 1 and 2 in all genes to those in the magenta and green‐yellow modules. We found that the mean GBM change in the magenta module was not significantly different than that of all other genes in the genome (*p* = 3.686, Figure 7). While we found that the mean GBM change was significantly different from all other genes in the genome, the effect size was negligible (−0.025, *p* = 1.053e‐07).

**FIGURE 8:**
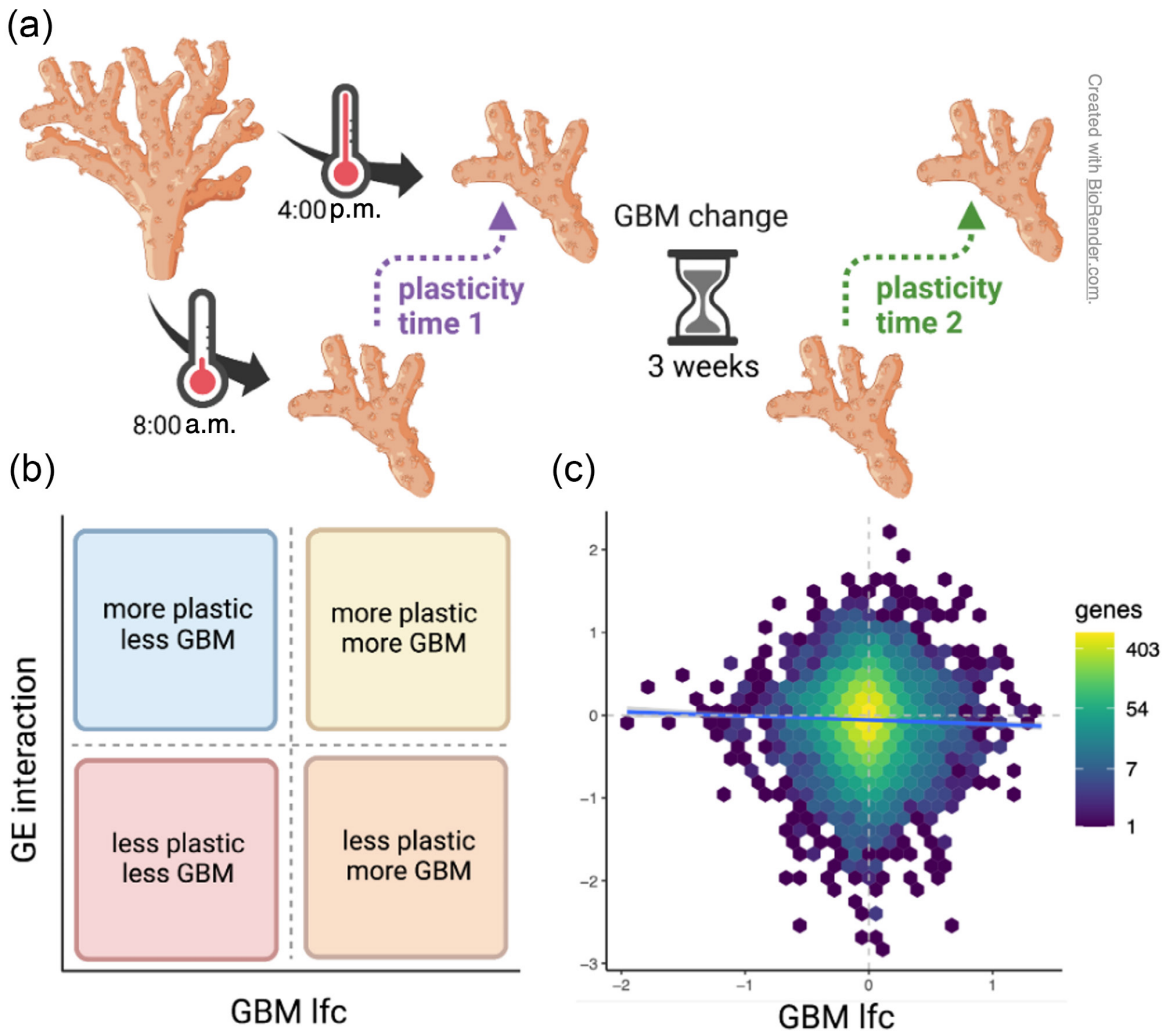
Correlation between change in daily plasticity and GBM change. (a) Experimental design showing the plasticity at timepoints 1 (purple) and 2 (green) between 8:00 a.m. and 4:00 p.m. (b) Hypothetical results showing the potential correlation between gene expression (effect of the interaction between the experimental timepoint and time of day) and GBM log_2_fold change. (c) Experimental results showing the actual correlation between the gene expression interaction term and GBM log_2_fold change (*r* ^2^ = 6.655e‐4, *p* = 0.004). Hexagons represent groups of genes binned together due to similar expression, and the color represents the number of genes in the hexagon. (Morning and afternoon samples).

We next included the afternoon samples to consider genes which were differentially expressed in the morning versus afternoon due to daily temperature fluctuations (Figure 8a). To measure the change in daily gene expression plasticity over the course of the experiment, we used the interaction term from our DESeq2 model, which represents the change in the magnitude of gene expression response to the afternoon temperature at the second timepoint vs. the first timepoint. A positive correlation between this change and GBM change would indicate that plasticity increased as GBM increased (yellow and red boxes in Figure 8b). On the other hand, a negative correlation between the two would indicate that plasticity increased as GBM increased (blue and orange boxes in Figure 8b).

Similar to the results from the treatment comparisons (Figure 6), we found a negligible correlation between change in daily plasticity over time and GBM change. This relationship was very weak, albeit significant, likely because of the large number of genes involved (Figure 8; *r* ^2^ = 6.655e‐4, *p* = 0.004).

## 4 DISCUSSION

Whether or not GBM plays a role in gene regulation in corals remains a matter of ongoing debate. It has been argued that gene body methylation (GBM) in invertebrates does not directly regulate gene expression since its change is neither necessary nor sufficient to induce gene expression change (Bewick et al., 2019; Cardoso‐Júnior et al., 2021; Harris et al., 2019). Furthermore, the correlation between GBM change and gene expression change is typically negligible or non‐existent (Dixon & Matz, 2022), which was also confirmed here (Figures 6 and 7). The primary objective of this work was to test whether environmentally driven changes in GBM are associated with changes in gene expression plasticity. We considered two groups of genes in heat‐acclimatized corals which retained heat‐state expression after being returned to the control tank (the green‐yellow and magenta modules, Figure 6e,f). However, acclimatization‐related GBM changes in these genes were either no different from the rest of the genes in the genome (magenta) or had a negligible effect size (green‐yellow, Figure 7). We also looked at genes which were differentially regulated between the coolest and hottest time of the day to see whether the magnitude of their daily plasticity change over time could be predicted by changes in GBM. We found no such association (Figure 8c).

If GBM does not play a role in acclimatization, then why does GBM change at all in response to the environment? It should be noted that this change is typically small compared to the change in gene expression: one study reported that even after transplanting corals across 4.5° of latitude for 2 months, their GBM remained mostly unchanged, with more than 80% of total GBM variation attributable to differences between original coral colonies (Dixon et al., 2018). Moreover, these changes consisted in genome‐wide increase or decrease of GBM disparity between the two broad gene classes, highly methylated and lowly methylated, rather than in targeted GBM adjustment in genes acting in specific pathways (Dixon et al., 2018). One way that environmental changes may alter broad GBM patterns is by affecting the expression and function of DNA methyltransferases. In vertebrates, changes in temperature are associated with altered expression and activity of DNMTs (Campos et al., 2012; Skjærven et al., 2014; Yan et al., 2015). In insects, it has been demonstrated that as little as a 4°C difference in temperature is correlated with changes in DNMT3 expression (Dai et al., 2018; Wang et al., 2020). If this is the case in corals, it is possible that GBM changes may simply be a consequence of spurious DNMT activity, which is consistent with our results (Figures 6 and 7).

Another possibility is that the microbiome may affect host epigenetic modifications. Corals are symbiotic organisms which harbor diverse, dynamic microbial communities (Putnam, 2021). Studies on human pathology have identified bacteria and viruses possessing proteins and enzymes, including DNMT homologs, which modulate host DNA methylation to facilitate infection (Bierne, 2017; Bierne et al., 2012; Kuss‐Duerkop et al., 2018). Using metagenomes of three coral species, one study identified candidate microbes which may produce similar proteins and enzymes (Barno et al., 2021). If this is the case, it is possible that shifts in the relevant abundance of bacteria in the coral microbiome may result in GBM changes in the host. However, experimental evidence for this theory in cnidarians remains lacking.

Another pattern that remains hard to explain is the association of GBM level with broad functional gene classes: in all multicellular animals that have any GBM at all, housekeeping genes show high GBM, and inducible (context‐dependent) genes show low GBM (Sarda et al., 2012). This could be a simple dynamic consequence of a more consistent transcription of housekeeping genes: after all, GBM is associated with transcriptional units, and it stands to reason that more consistently transcribed genome regions would attract more DNA methylation activity, likely to prevent intragenic transcription initiation (Neri et al., 2017). However, if transcription is the only GBM distribution determinant, exons should not be different from introns, yet GBM is almost always higher in exons relative to introns (Liew et al., 2018; Singer et al., 2015; Wang et al., 2013). Assuming that GBM marks are at least partially heritable (Liew et al., 2020; Wang et al., 2013), this raises an interesting possibility that GBM affects gene function, and its distribution in the genome might be shaped in part by natural selection. If so, from an evolutionary standpoint, GBM may be acting as a genetic mutation process, introducing heritable (and perhaps idiosyncratic) perturbations into gene function and subject to natural selection. We therefore believe that studying GBM in a population genetic /adaptation framework (rather than gene regulation /acclimatization framework) is warranted to see if GBM may be the source of adaptive, genetic variation in natural populations.

## CONFLICT OF INTEREST STATEMENT

The authors have no conflicts of interest related to this publication.

## Supporting information


Figure S1.
Figure S2.
Figure S3.
Figure S4.

## Data Availability

All scripts used in this analysis are available at https://github.com/evelynabbott/DNA_methylation_plasticity. Raw TagSeq and MdRAD reads can be accessed under the NCBI BioProject accession PRJNA1003549. The *Acropora millepora* genome is available on the Matz Lab website, https://matzlab.weebly.com/data‐‐code.html.
