# Supplementary material for: Modifications to gene body methylation do not alter gene expression plasticity in a reef‐building coral
Source: Evol Appl. 2024 Feb 22;17(2):e13662. doi: 10.1111/eva.13662 (PMC10883760; doi:10.1111/eva.13662)
Supplement: Supplementary file 1 — Figure S1. Figure S2. Figure S3. Figure S4. [file EVA-17-e13662-s001.docx]

**Supplemental materials**

Figure S1.


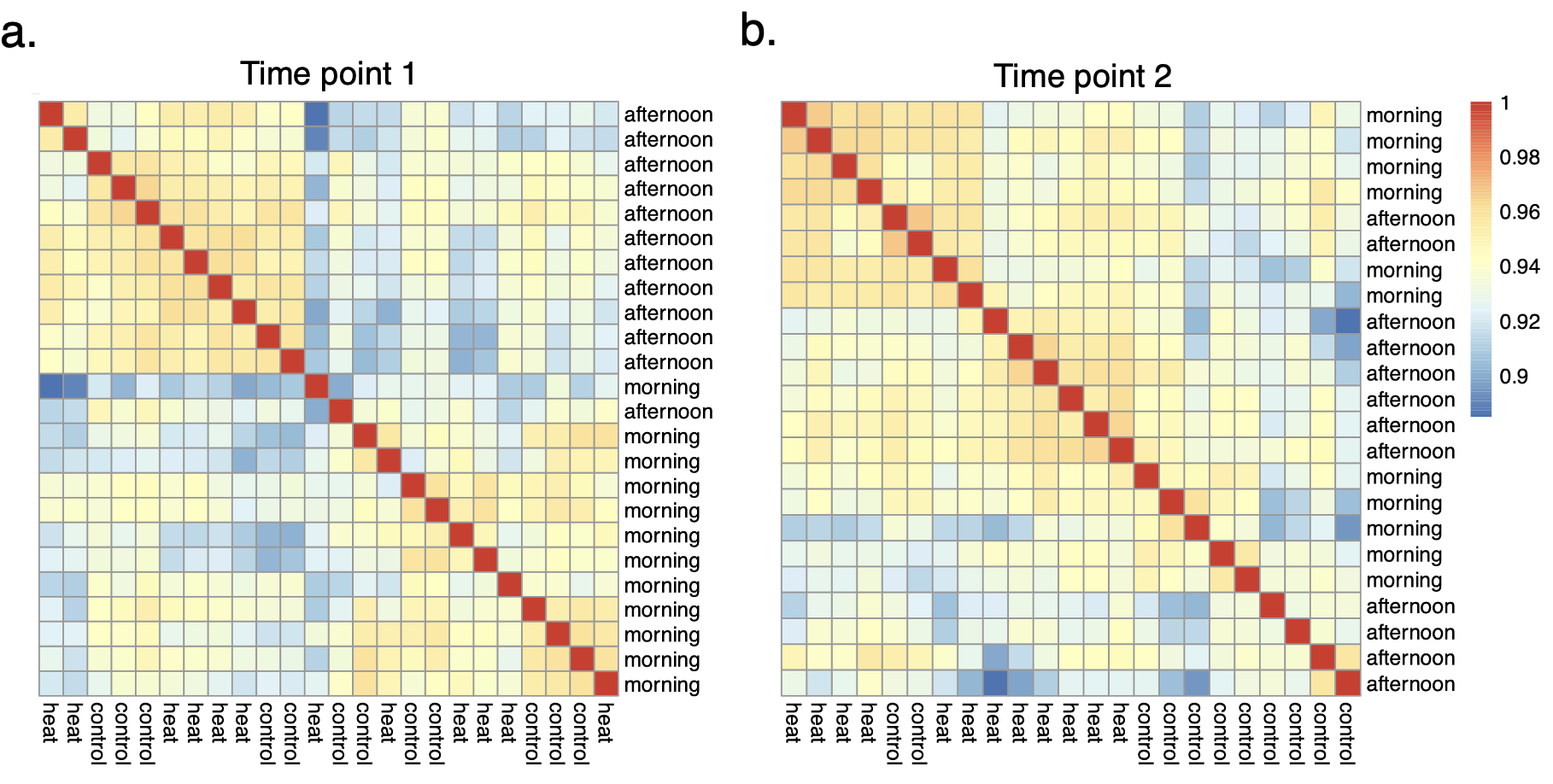


Figure S1. Broad patterns of gene expression in heat and control groups in the morning and afternoon at time point (a) and time point 2 (b). Heatmaps show similar (red) and different (blue) gene expression between samples.

Figure S2.


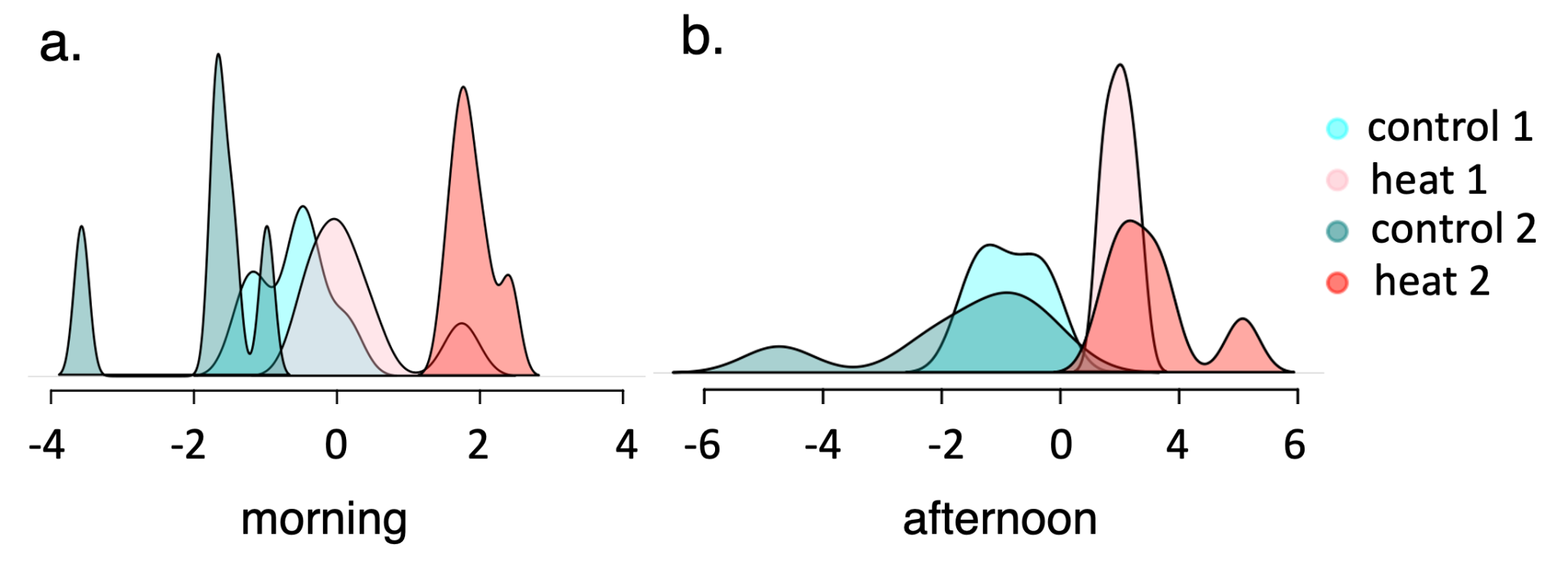


Figure S2. Discriminant analysis of principal components (DAPC) plotted for each time point in the morning (a) and afternoon (b). Colors represent control treatments (light teal for time point 1, dark teal for time point 1) and heat treatments (pink for time point 1, red for time point 2).

Figure S3.


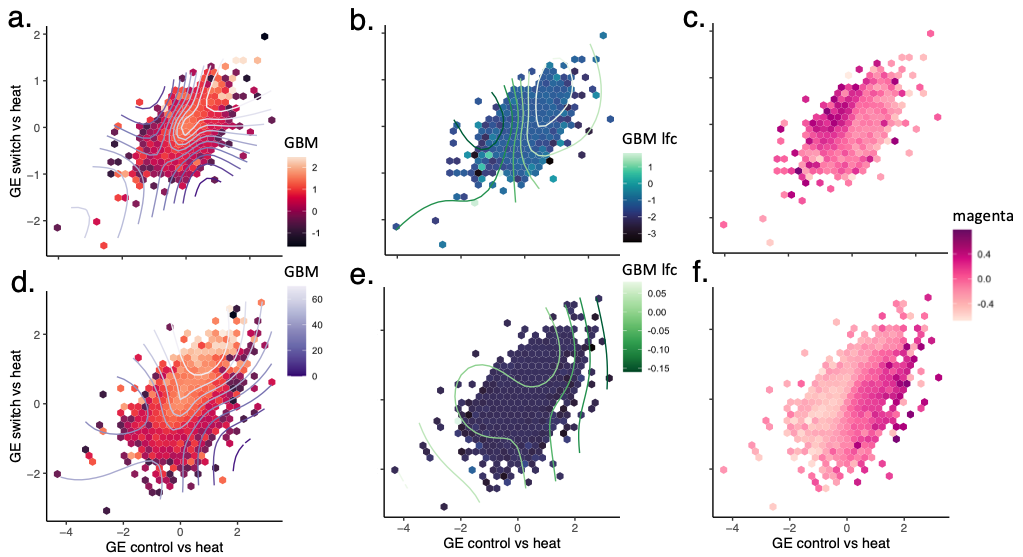


**Figure S3.** Experimental results showing correlation between control and switch gene expression at the end of the experiment, including afternoon samples (a-c) and excluding morning samples (d-f). Hexagons represent groups of genes binned together due to similar expression. a-c) Including all samples, the color of the hexagons and contour lines correspond to GBM basemean (a, r^2^ = 0.041, p <2e-16), GBM lfc (b, r^2^ = -5.61e-08, p = 0.719), and membership in the magenta module (c). d-f) Including only afternoon samples, the color of the hexagons and contour lines correspond to GBM basemean (d, r^2^ = 0.084, p <2e-16), GBM lfc (e, r^2^ = 0.003, p = 7.59e-06), and membership in the magenta module (f).

**Figure S4.**

**
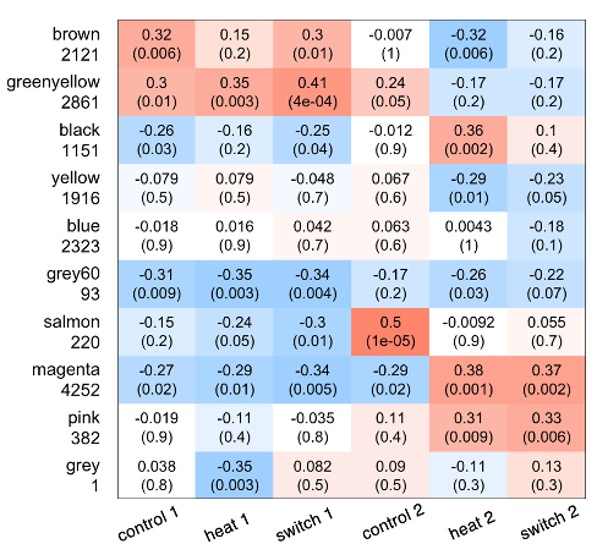
**

**Figure S4.** Figure 3. Expression of WGCNA modules in all treatment groups (control, heat, or switch) and time points (1 or 2). Red indicates upregulation and blue indicates downregulation. Row names correspond to module names and the number of genes in the module. The numbers in each cell represent the significance of module expression in each treatment group (top is R, bottom is p value).
